# Circulating adhesion molecules and arterial stiffness

**DOI:** 10.5830/CVJA-2014-060

**Published:** 2015

**Authors:** Ismail Dogu Kilic, Yusuf I Alihanoglu, Bekir Serhat Yildiz, Harun Evrengul, Gulin Findikoglu, Sukriye Uslu, Simin Rota

**Affiliations:** Department of Cardiology, Medical Faculty, Pamukkale University, Denizli, Turkey; Department of Cardiology, Medical Faculty, Pamukkale University, Denizli, Turkey; Department of Cardiology, Medical Faculty, Pamukkale University, Denizli, Turkey; Department of Cardiology, Medical Faculty, Pamukkale University, Denizli, Turkey; Department of Physical Medicine and Rehabilitation, Cardiopulmonary Unit, Medical Faculty, Pamukkale University, Denizli, Turkey; Finike State Hospital, Antalya, Turkey; Department of Biochemisty, Medical Faculty, Pamukkale University, Denizli, Turkey

**Keywords:** VCAM-1, ICAM-1, adhesion molecules, aortic stiffness

## Abstract

**Aim:**

VCAM-1 and ICAM-1 are two important members of the immunoglobulin gene superfamily of adhesion molecules, and their potential role as biomarkers of diagnosis, severity and prognosis of cardiovascular disease has been investigated in a number of clinical studies. The aim of the present study was to determine the relationship between circulating ICAM-1 and VCAM-1 levels and aortic stiffness in patients referred for echocardiographic examination.

**Methods:**

Aortic distensibility was determined by echocardiography using systolic and diastolic aortic diameters in 63 consecutive patients referred for echocardiography. Venous samples were collected in the morning after a 12-hour overnight fast, and serum concentrations of ICAM-1 and VCAM-1 were measured using commercial enzyme immunoassay kits.

**Results:**

Data of a total of 63 participants (mean age 55.6 ± 10.5 years, 31 male) were included in the study. Circulating levels of adhesion molecules were VCAM-1: 12.604 ± 3.904 ng/ml and ICAM-1: 45.417 ± 31.429 ng/ml. We were unable to demonstrate any correlation between indices of aortic stiffness and VCAM-1 and ICAM-1 levels.

**Conclusion:**

The role of soluble adhesion molecules in cardiovascular disease has not been fully established and clinical studies show inconsistent results. Our results indicate that levels of circulating adhesion molecules cannot be used as markers of aortic stiffness in patients.

## Abstract

Damage to or stimulation of the endothelium leads to the increased expression and release of molecules that trigger leukocyte homing, adhesion and migration into the subendothelial space, which are fundamental stages of the development and progression of atherosclerosis.^1^ Among these, adhesion molecules play a key role. Adhesion molecules are substances that mediate the interaction between cells, their extracellular matrices and endothelial surfaces. They function as receptors that trigger intracellular pathways and participate in the control of vital processes.^2^

Once expressed on the endothelial surface, soluble forms of adhesion molecules may be found in the circulation, released either via shedding or proteolytic cleavage, and are considered markers of increased expression of membrane-bound adhesion molecules.^3^-^5^ Vascular cell adhesion molecule 1 (VCAM-1) and intercellular adhesion molecule 1 (ICAM-1) are two important members of the immunoglobulin gene superfamily of adhesion molecules and their potential role as biomarkers of diagnosis, severity and prognosis of cardiovascular disease has been investigated in a number of clinical studies.^6^

Decreased arterial compliance is one of the earliest signs of adverse structural and functional changes within the vessel wall.^7^ Increased arterial stiffness represents a physiological aspect of ageing, however, this process can be accelerated by cardiovascular risk factors, and has been shown to be an independent predictor of cardiovascular morbidity and all-cause mortality in various populations.^8^-^10^

Although studies have also demonstrated that increased arterial stiffness is associated with inflammation, data on the association between aortic distensibility and soluble adhesion molecules are sparse.^11^ The purpose of the present study was to determine the relationship between circulating ICAM-1 and VCAM-1 levels as inflammatory markers, and aortic stiffness in patients referred for echocardiographic examination.

## Methods

Sixty-three consecutive patients who were referred for echocardiography were included in the study. Patients with renal or hepatic failure, known infectious or inflammatory disease, acute illness, moderate-to-severe valvular dysfunction, aortic dissection or other aortic disease, or poor acoustic quality were excluded. The study was approved by our local ethics committee, and written informed consent was obtained from each participant.

Aortic stiffness measurements were performed on the subjects in the left lateral decubitus position with echocardiography, using a Vivid 7 Doppler echocardiographic unit (GE Vingmed Ultrasound, Horten, Norway) with a 2.5-MHz probe. The aortic diameter was recorded by M-mode echocardiography at a level of 3 cm above the aortic valve.

Internal aortic diameters were measured by means of a caliper in systole and diastole as the distance between the trailing edge of the anterior aortic wall and the leading edge of the posterior aortic wall. Aortic systolic (AoS) diameter was measured at the time of full opening of the aortic valve, and diastolic (AoD) diameter was measured at the peak of QRS. Three consecutive beats were measured routinely and averaged.

Systolic and diastolic blood pressures were measured simultaneously at the brachial artery by sphygmomanometry. Pulse pressure was calculated as systolic minus diastolic blood pressure.

The percentage change of the aortic root Ao (%) was calculated to obtain the aortic strain:

$Ao\;\left(\%\right)=\;\frac{100\;\times (AoS\;-AoD)}{AoD}$

Other indices of the aortic elastic properties were measured.

Aortic distensibility index (cm^-2^ dyn^-1^ 10^-6^)

$=\;\frac{2\;\times (systolic\;diameter\;-diastolic\;diameter)}{\left(diastolic\;diameter\right)\;\times (pulse\;pressure)}$

$The\;aortic\;stiffness\;index\;\beta =\;\frac{1n(\frac{SBP}{DBP})}{(AoS\;-AoD)/AoD}$

All venous samples were collected in the morning after a 12-hour overnight fast, for biochemical analyses. The blood samples were centrifuged at 4 000 rpm at room temperature for 5 min, and the plasma was frozen at –20°C until measurement of adhesion molecules. Serum concentrations of ICAM-1, and VCAM-1 were measured using commercial enzyme immunoassay kits (E-BIOSCIENCE, San Diego, USA), as instructed by the manufacturer.

## Statistical analysis

All data analyses were performed with the SPSS (Statistical Package for Social Sciences) for Windows 17.0 computer program (SPSS Inc. Chicago, IL, USA). Data were expressed as mean ± standard deviation. After testing for normality with the Shapiro–Wilk test, continuous parameters were analysed with non-parametric tests. The relationship between levels of circulating adhesion molecules and aortic stiffness was assessed by Spearman’s test. Since preliminary analysis did not reveal significant interactions with cellular adhesion molecules, we did not run models stratified by risk factors for aortic stiffness. A *p*-value of < 0.05 was accepted as significant.

## Results

Data from a total of 63 participants (mean age 55.6 ± 10.58 years, 31 male) were included in the study. The baseline characteristics of the study population are summarised in Table 1. Circulating levels of adhesion molecules were VCAM-1: 12.604 ± 3.904 ng/ml and ICAM-1: 45.417 ± 31.429 ng/ml. Aortic strain was 6.210 ± 2.253%, stiffness index was calculated as 10.423 ± 5.350 and distensibility as 2.354 ± 0.993 × 10^-3^ /KPa (Table 2). We were unable to demonstrate any correlation between the indices of aortic stiffness and CAM-1 and ICAM-1 levels (Table 3).

**Table 1:** Baseline characteristics of the patients

*Characteristics*	*Number (%)*
Age (years)	55.6 ± 10.5
Males	31 (49.2)
Smoking	6 (9.5)
Hypertension	31 (49.2)
Diabetes mellitus	14 (22.2)
Hyperlipidaemia	11(17.5)
Coronary artery disease	18 (25.6)

**Table 2:** Serum VCAM and ICAM levels and indices of aortic stiffness

*Variables*	*Mean ± SD (n = 63)*
VCAM (ng/ml)	12.604 ± 3.904
ICAM (ng/ml)	45.417 ± 31.429
Aortic strain (%)	6.210 ± 2.253
Stiffness index (β)	10.423 ± 5.350
Distensibility (× 10^-3^/KPa)	2.354 ± 0.993

**Table 3:** Correlation between aortic strain, stiffness index, distensibility and adhesion molecules

*Variables*	*VCAM (ng/ml)*		*ICAM (ng/ml)*	
	*r*	*p*	*r*	*p*
Aortic strain (%)	–0.030	0.813	0.061	0.634
Stiffness index (β)	0.038	0.768	–0.095	0.458
Distensibility (× 10-3/KPa)	–0.026	0.839	0.097	0.449

## Discussion

The potential role of soluble adhesion molecules as biomarkers of diagnosis, severity and prognosis of cardiovascular disease have been investigated in a number of clinical studies. However, these studies have found heterogeneous results. Ridker *et al.* reported a significant association between increasing concentrations of sICAM-1 and the risk of future myocardial infarction, especially among participants with baseline sICAM-1 concentrations in the highest quartile.^12^

Blankenberg *et al.* found that VCAM-1, ICAM-1 and E-selectin were significantly related to future cardiovascular death in 2.7 years’ mean follow up of a prospective cohort of 1 245 patients.^3^ Moreover, of all the inflammatory markers evaluated, VCAM-1 levels revealed the strongest association with future death, and added predictive value to the classic risk factors and high-sensitivity C-reactive protein (CRP) in determining the risk for future cardiovascular death.

In a prospective, nested, case–control study, median levels of sICAM-1 but not sVCAM-1 were significantly higher at baseline among men who developed peripheral arterial disease (PAD) during a nine-year follow-up period.^13^ In the study by Hwang *et al.*, E-selectin and ICAM-1 levels were significantly increased in patients with coronary heart disease (CHD) and carotid artery atherosclerosis compared with the control subjects. However, levels of VCAM-1 were not significantly different among patients in these groups.^14^

In contrast to these findings, in a long-term, communitybased study, Malik *et al.* assessed the predictive ability of baseline serum concentrations of soluble adhesion molecules for fatal and non-fatal CHD. They found no strong association of these adhesion molecules with CHD risk. Furthermore, they reinforced their findings with a meta-analysis of previously published prospective studies.^15^

Whether soluble adhesion molecules are increased in subclinical cardiovascular disease has been investigated in several studies. In a sample from the Monitoring Trends in and Determinants in Cardiovascular Disease (MONICA) trial, despite sICAM-1 levels being independently associated with the risk of having at least one carotid or femoral plaque, no significant association was found with carotid intima–media thickness (CIMT).^16^ Similarly, Amar *et al.* showed interleukin-6 and ICAM levels were associated with stable atherosclerotic plaque but not with IMT.^17^

In a prospective study by Gross *et al.*, higher sICAM1 levels were associated significantly and in a graded fashion with common CIMT in participants with advanced plaque.^18^ The study indicated an early (mean age 40 years) involvement of sCAM1 in the development of atherosclerosis, independent of traditional cardiovascular risk factors and CRP levels. Moreover, no association was found in patients with low total burden of atherosclerosis.

Our results are in alignment with these studies. In consecutive patients referred for echocardiographic examination, we found no association between adhesion molecules and aortic stiffness, which is a predictor of cardiovascular disease.

Despite all these studies, the role of soluble adhesion molecules in cardiovascular disease has not been fully established and clinical studies show inconsistent results. There are some possible explanations for this inconsistency. First, vascular endothelial and smooth muscle cells express VCAM-1, while ICAM-1 expression is not limited to these cells and is expressed in many cells, including haematopoietic cells and fibroblasts.^3^ Therefore, VCAM-1 may be a marker of plaque burden or activity, whereas ICAM-1 may be a marker of low-grade inflammation. Some authors have suggested that ICAM-1 is predictive in initially healthy people and VCAM-1 in patients with atherosclerosis.^3^

Second, levels of soluble adhesion molecules are influenced by age, smoking status, diabetes and other inflammatory conditions, and even with exercise or changes in arterial pressure.^19^,^20^ Moreover, there is a lack of knowledge of which cellular and molecular factors determine the levels of adhesion molecules, since VCAM and ICAM, like other inflammatory molecules, may have regulations at many levels.^6^

Aortic stiffness is an independent predictor of cardiovascular risk. Arterial stiffening is a physiological aspect of ageing and is the result of the joint effects of adhesion molecules, integrins, metalloproteinases, the renin–angiotension system, and inflammation of cellular components (endothelium, vascular smooth muscle, fibroblasts and matrix components) on the structural and functional properties of the artery.^21^ Indeed, recent studies have shown the importance of inflammation in arterial stiffening. Increased levels of inflammatory markers have been associated with arterial stiffness in various groups, including healthy subjects, hypertensives, and community-based groups.^21^-^25^

In the study of Bussel *et al.*, biomarkers of endothelial dysfunction and low-grade inflammation, including adhesion molecules, were associated with greater arterial stiffness over a six-year period.^26^ A causative effect of acute systemic inflammation on increasing large-artery stiffness and decrease in wave reflections was also shown in patients receiving vaccinations.^27^

Several mechanisms may explain the link between arterial stiffness and inflammation. First, degradation of the elastin and collagen of the vessel wall may be increased by activation of matrix metalloproteinases, which may be mediated by increased levels of inflammatory mediators, including adhesion molecules.^11^ Inflammation may also provoke fibrosis and smooth muscle proliferation, which would subsequently cause arterial stiffness.^11^

Another possible explanation is the major role of the endothelium in arterial stiffness. Inflammation causes endothelial dysfunction and alters arterial distensibility by impairing the production of vasodilatory factors.^27^ One last, speculative explanation could be arterial stiffness causing inflammation, since elevated pulse pressure and increased shear stress may stimulate inflammation and increase the expression of adhesion molecules.^20^,^22^,^28^

## Conclusion

We were unable to find a significant correlation between aortic stiffness and circulating adhesion molecules (VCAM-1 and ICAM-1). Possible causes of this finding have been discussed above. However, other factors may have affected the results. The mean age in our study was 55.6 ± 10.5 years. Since arterial stiffening typically occurs after the age of 60 years, our study population represented a relatively young population.^26^ Second, arterial stiffening is not a uniform condition in all arterial systems, and aortic distensibility is a local measure of arterial stiffness.^22^ Third, levels of adhesion molecules were measured only once, and they may be subject to intra-individual variability. The small study size is another limitation of this study.
